# The Use of Sedatives in Phthisis

**Published:** 1896-01-25

**Authors:** Solomon C. Smith

**Affiliations:** Physician to the Westminster General Dispensary.


### THE USE OF SEDATIVES IN PHTHISIS.

By SOLOMON C. SMITH, M.D., M.R.C.P., Physician to the Westminster General Dispensary.

THERE is among many physicians a strong feeling against the use of sedatives for the relief of some of the distressing accompaniments of phthisis. It is said that they, and the preparations of opium especially, upset the stomach, disturb the appetite, interfere with the action of the bowels, and thus, while easing present distress, merely smooth the patient's path downwards to the grave. In this there is some truth, and there can be no doubt that the use of sedatives for the treatment of symptoms which admit of effectual relief by other means is a mistake. Often and often it is the case that painting the pharynx with solution of tannin, or gargling with an alkaline lotion, or the simple sipping of a mixture containing bicarbonate of soda and some carminative, will relieve a cough which at first sight may appear to demand an opiate linctus, and there can be no doubt that the rough-and-ready treatment of cough by morphia syrups and lozenges, to be swallowed all day long, is injurious in the extreme; but, on the other hand, one must not allow the dread of disturbing the digestive organs to stand in the way of the administration of opium and its preparations in cases where they are really wanted, as they so often are in the disease under consideration.

The great use of sedatives in phthisis is for the relief of cough. The obviousness of the facts that cough is necessary to bring up expectoration, and that expectoration gives more relief than anything else leads sometimes to the irritating advice that the cough had better be let alone, a form of counsel which more than any other drives consumptive patients into the hands of quacks. A constant barking cough is absolutely maddening to some people, and the temptation offered by the syrups and elixirs on the chemist's counter irresistible. It must be remembered too that there is but one good thing that cough can do, and that is to bring up expectoration. Everything else that it does is evil; it irritates the throat, it injures the larynx; it strains the injured lung; it disturbs the stomach, often causing the rejection of food, which, but for it, might have been digested; and it produces fatigue even to utter exhaustion. The aim, then, of all treatment directed to the cough of phthisis should be to stop it—on the one condition that expectoration from the lung shall not be interfered with.

Practically all cough is reflex, and, putting on one side laryngeal tuberculosis, it may be broadly stated that phthisical cough is set up by irritation in the pharynx, in the stomach, in the air tubes, or in the diseased spot. Now, in regard to the first two of these points of origin, we need have no hesitation in stopping it by every means at our command, without regard to expectoration, first of all by removing the irritation, but if that fails, then by sedatives. The indigestion of consumption not only aggravates the disease, but is often the origin of much of the cough which accompanies it, and, before giving sedatives for a teasing cough, it is often well to try alkalies, bismuth, rhubarb, bitters, and carminatives, and even, if the stomach is known to be full and sour, an emetic or a stomach tube.

Again, the pharynx is a common starting-point of cough, which does no good, and should, therefore, be stopped without hesitation—not always, however, by sedatives. Often consumptive patients, of gouty tendency, are, by the diet ordered for the one complaint, brought within an ace of the other, and develop a pharyngitis which is, to all intents and purposes, a form of gout, and is best relieved by potash and colchicum or by salicylate of soda with ammonia. But even if this is not the case, it may happen that much adhesive mucus lines the pharynx, in which case it should be removed by the brush or by gargles before sedatives are given, for a vastly smaller quantity of morphine allowed to trickle down a well-cleaned throat will give relief than would be required if the accumulated mucus were left untouched. A very great deal of the cough of phthisis is the result of the accompanying bronchitis, and it is necessary to make use of the ordinary remedies for this condition before reverting to those required for merely stopping cough. This is the reason why it is best not, as a rule, to add sedatives to expectorants. Ammonia, squills, senega, ipecacuanha, antimony, and apomorphine are all useful, and when their effects have been produced, then sedatives may be given to abate the residual irritation; but as a general rule to combine sedatives with expectorants is a mistake, it being better to produce a free expectoration, and then when the swelling and congestion is relieved to soothe the irritation. It may, however, be impossible to wait so long, the patient may be exhausted with the cough and sleep may be necessary, in which case it is best to give a sufficient dose of morphine or chloral to stop the cough rather than to soothe it, and it will not infrequently be found that during the period of quiescence the phlegm has "matured" and so separated itself from the underlying membrane as to be expelled with unexpected ease.

The same idea must underlie our treatment of the cough which arises from the accumulation of fluid in cavities. Cough often comes on when patients lie down, apparently from the altered position assumed by the fluid contents of cavities, and no relief occurs until expectoration has taken place; under such circumstances a sedative, which patients often pray for, is ridiculous. The first thing is to obtain an emptying of the cavity by lying in that position which experience teaches is most sure to bring on the cough, and then when expectoration has occurred to give such sedative as may get sleep.

It may then be taken as a broad rule that in the treatment of phthisical cough sedatives should not be mixed with cough mixtures to be taken frequently, but that when required they should be given in sufficient doses, not merely to mitigate, but for the time to cure cough, and so give a period of rest, and that before giving them all proper measures should be taken to remove reflex causes of irritation.

